# Interocular Asymmetry of Foveal Thickness in Parkinson Disease

**DOI:** 10.1155/2012/728457

**Published:** 2012-08-01

**Authors:** Eric M. Shrier, Christopher R. Adam, Brian Spund, Sofya Glazman, Ivan Bodis-Wollner

**Affiliations:** ^1^Department of Ophthalmology, SUNY Downstate Medical Center, State University of New York, 450 Clarkson Avenue, Brooklyn, NY 11203, USA; ^2^SUNY Eye Institute, Brooklyn, NY 11023, USA; ^3^College of Medicine, SUNY Downstate Medical Center, State University of New York, 450 Clarkson Avenue, Brooklyn, NY 11203, USA; ^4^Department of Neurology, SUNY Downstate Medical Center, State University of New York, 450 Clarkson Avenue, Brooklyn, NY 11203, USA

## Abstract

*Purpose*. To quantify interocular asymmetry (IA) of foveal thickness in Parkinson disease (PD) versus that of controls. *Design*. Prospective case-control series. *Methods*. *In vivo* assessment of foveal thickness of 46 eyes of 23 PD patients and 36 eyes of 18 control subjects was studied using spectral domain optical coherence tomography (SD-OCT). Inner versus outer layer retinal segmentation and macular volumes were quantified using the manufacturer's software, while foveal thickness was measured using the raw data from each eye in a grid covering a 6 by 6 mm area centered on the foveola in 0.25 mm steps. Thickness data were entered into MATLAB software. *Results*. Macular volumes differed significantly at the largest (Zone 3) diameter centered on the foveola (ETDRS protocol). By segmenting inner from outer layers, we found that the IA in PD is mostly due to changes on the slope of the foveal pit at the radial distances of 0.5 and 0.75 mm (1.5 mm and 1 mm diameter). *Conclusions*. About half of the PD patients had IA of the slope of the foveal pit. IA is a potentially useful marker of PD and is expected to be comparable across different SD-OCT equipment. Data of larger groups may be developed in future multicenter studies.

## 1. Introduction

Parkinson disease (PD) predominantly affects motor functions, but nonmotor deficits in PD have attracted interest as potential diagnostic and treatment biomarkers. 

PD patients commonly have subjective visual difficulties that are not well understood. Spectral domain optical coherence tomography (SD-OCT) allows quantification of the thickness of the fovea, the anatomical site of most acute vision, and is emerging as a potential tool in PD [1]. A possible causal link between common visual complaints experienced in PD and retinal dysfunction is supported by the presence of dopaminergic neurons (amacrine cells) [2, 3] of the healthy human adult retina and their impairment in PD [4–6]. 

*In vivo* evidence of manifest retinal impairment in PD emanated from a number of retinal electrophysiologic studies, using the pattern electroretinogram (PERG) which suggested that the retina is the most distal source of visual impairment in PD [7–14] (see Supplementary Material 1 available online at doi:10.1155/2012/728457).

SD-OCT retinal scanning is notably fast, readily available, reproducible, noninvasive, and inexpensive as a candidate biomarker. It also supplies a near-histopathologic image of the retina, *in vivo* (see Supplementary Material 2).

Using OCT, it was first shown in 2004 [15] that the nerve fiber layer (NFL) of the retina is thinned in PD. Subsequently retinal thinning in PD was confirmed in most studies [16–19], although details and diagnostic yield differed. There may be several reasons. Most of the studies concentrated on the NFL and not on the inner, cellular retina or they have averaged across the entire fovea and computed total “macular volumes,” thereby reducing the likelihood of finding significant differences of diverse retinal layers.

 In this study, we concentrated on the foveal pit, where different retinal layers are easier to separate. It is also essential that the comparison control group excluded subjects with presenile dementia and early neurodegenerative conditions, which happen to predominantly occur in the aged. Furthermore, differences exist in the selection of “number of eyes studied versus number of subjects” [20]. Finally, in the statistical analysis of ophthalmic data, one has to account for a correlation between the two eyes [21]. Examination of IA reduces the influence of the natural variation in the thickness of retinal layers. We report significant IA of perifoveolar thickness of the preganglionic retina in PD.

## 2. Methods

### 2.1. Subjects

This was a prospective case-control clinical series. The study was approved by the Institutional Review Board for Human Subjects Research of SUNY Downstate Medical Center, and the study adheres to the tenets of the Declaration of Helsinki. Both groups were examined using identical comprehensive neurological and ophthalmological examinations (see Supplementary Material 3). All subjects had best-corrected Snellen visual acuity better than 20/30. There were 46 PD eyes (23 patients) and 36 consecutive age-matched control eyes (18 subjects). PD patients were diagnosed based on the UK Brain Criteria [23]. They were staged using the Hoehn and Yahr (H-Y) [24] criteria and scored according to the standardized clinical tests of UPDRS. The mean ages of PD patients and of healthy subjects were 64.6 ± 7.5 (SD) versus 61.5 ± 9.0 years (*P* = 0.77). The mean H-Y stage was 3.2 (range 1–4) for PD subjects.

As defined by the makers of the SD-OCT software, the measurement of the inner retinal layer (IRL) includes internal limiting membrane (ILM), nerve fiber layer (NFL), ganglion cell layer (GCL), and inner plexiform layer (IPL). Amacrine cells, including dopaminergic amacrine cells, are located in the inner nuclear layer at the border of the IPL. The outer retinal layer (ORL) includes layers from the inner nuclear layer, Henle's fiber layer (HF), outer nuclear layer (ONL), inner (IS) and outer (OS) segments of the photoreceptors up to the retinal pigment epithelium (RPE). full retinal thickness (FRT) is measured from the ILM to the RPE.

### 2.2. OCT Methodology

**Figure 1:**
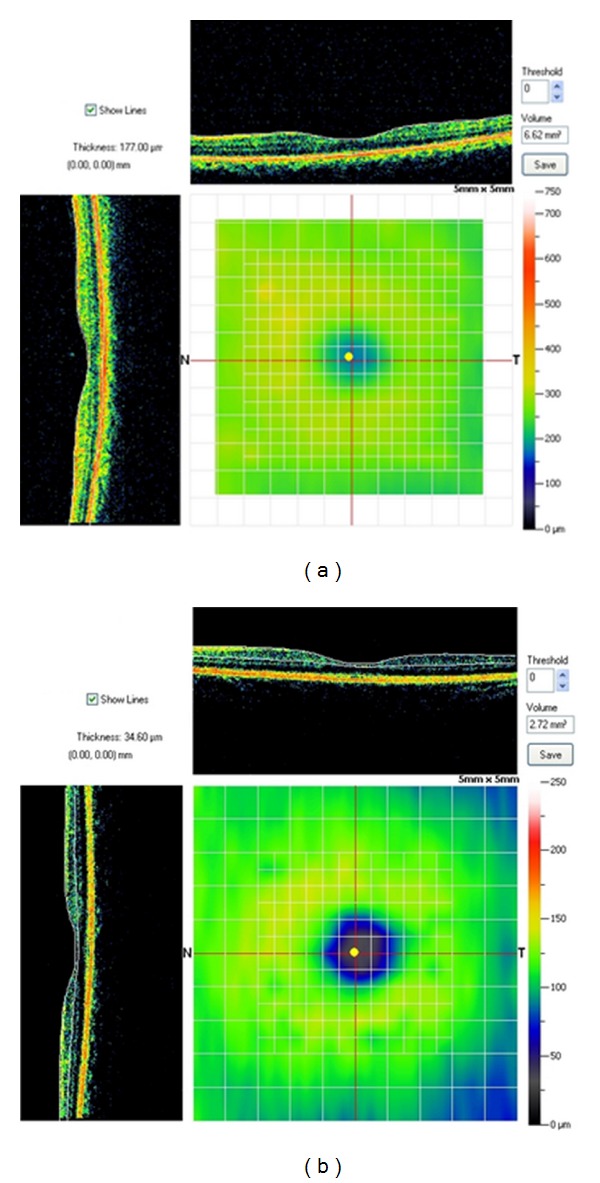
The central yellow dot in this illustration represents fixation at the foveola. The measuring grid visible in the background is centered on the foveola. Thicknesses at each of the intersecting points of the grid and calculated the volume of each 0.25 × 0.25 mm voxel. (a) shows the SD-OCT profiles through the fovea for a control subject and (b) SD-OCT profile for a PD patient.

Participants were scanned with high resolution SD-OCT (RTVue Model RT 100 (Optovue, Inc.; Fremont, CA)). Grids of 5 × 5 mm (MM5 scan protocol) or 6 × 6 mm (EMM5 scan protocol) are automatically placed to depict, map, and measure sections of the retina. The grids are centered on the area of visual fixation, the foveola (yellow dot) (see Figure 1(a) (control) and Figure 1(b) (PD)). Centration, apparent registration of each scan, and the qualitative regularity of automatically applied segmentation lines were checked for each scan. The program automatically segments inner and outer retinal layers, applies three lines, and quantitative data is then internally computed to yield inner and outer retinal layer and full-thickness retinal measurements. The corresponding numerical data is then exported for statistical analysis.

We manually placed the cursor at each of the intersecting points of the grid and calculated the volume of each 0.25 × 0.25 voxel by the data provided in the OCT equipment. This (mathematically) yields a matrix of 401 elements per eye studied. Only scans that are of sufficient quality (signal strength = 75% of maximal strength, absent unwanted imaging artifacts, or distortions) were accepted. Images obtained when the vertical and horizontal scans were displaced by more than one voxel (0.25 mm) were rejected.

Macular volumes were quantified using the software of the manufacturer based on the ETDRS protocol. These values are taken off from the automated program of the RT-Vue. They allowed a comparison for foveal thickness and three retinal volumes centered on the foveola with radii of 0.5, 1.5, and 3 mm. 

Quantifying segmented foveal thickness: based on the results of our prior study [1] we examined interocular, within-subject variability of the perifoveal area in PD. Inner retinal thickness was measured from the ILM up to and including the boundary interface of the IPL and INL. We exported the corresponding OCT data (images were not manipulated) into MATLAB.

### 2.3. OCT Interocular Difference (IA), Statistical Analysis

The statistical analysis was performed for both macular volume and segmented retinal thickness (inner versus outer). All statistical analyses were performed using PASW release number 19. The general linear model for repeated measures was used to test for main effect differences between the control and Parkinsonian groups, race, changes over foveal zones, and the interaction of group by foveal zones. Mauchly's test for sphericity was significant indicating a violation of equality of variances between the interocular differences over foveal zones. The Greenhouse-Geisser correction was applied to correct for this violation. For comparing IA at distinct perifoveolar radial distances, the thickness difference for each corresponding voxel was calculated for the four cardinal directions between the left and the right eyes of each subject.

## 3. Results 

### 3.1. Foveal Thickness and Macular Volumes

Central foveal thickness (0 mm distance = foveola) was the same for PD subjects and controls. There was no effect for group (*F* = 0.07, *P* = 0.79) or for interaction of group by race (*F* = 0.78, *P* = 0.47). 

**Figure 2:**
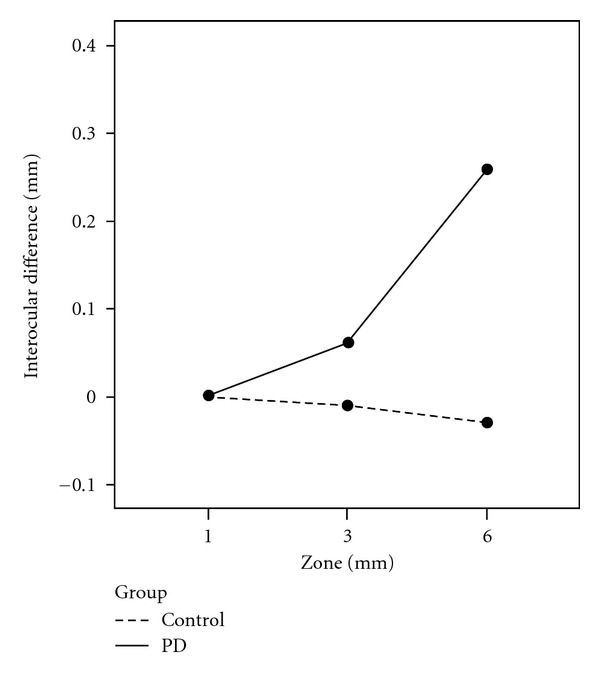
This figure shows interocular asymmetry in the average macular volume measured in the three “standard” (ETDRS) peri-foveal zones. The difference increases with zone diameter (5 mm) and reaches statistical significance only when the total macular volume (Zone 3) is compared (see Methods) between PD and control subjects.

There was significant difference (*F* = 4.32, *P* = 0.046) between the groups in macular volume over the largest diameter (Zone 3 of the ETDRS protocol) (see Figure 2). 

This difference in total macular volume is consistent with the results reported by Altintas et al. [16]. There was no effect for the difference between races (*F* = 0.341, *P* = 0.713) or group by race (*F* = 0.144, *P* = 0.87). After correction, there was no significant difference for changes in foveal zone (*F* = 1.55, *P* = 0.22) or the interaction of foveal zone by group (*F* = 2.38, *P* = 0.13). 

### 3.2. Segmented IRL Thickness Measurements

the absolute value of the interocular difference (IOD) was calculated for each control subject and PD patient for each measured radial distance from the foveola. In the PD group, mean IOD reached 12.26 microns at 1.00 mm perifoveolar radius, while, at the corresponding distance controls had a mean 6.97 micron IOD. The mean IOD by group and 1 SD for each distance in controls are shown in Table 1.

At each radial distance, every eye's IA in the four directional quadrants was averaged, and a mean quadratic thickness value was obtained.

The number and percentage of control subjects whose interocular difference was greater than control means at 1 SD are shown in Table 2. Tables 3 and 4 depict the same derived measures for 1.5 and 2 SD.

**Figure 3:**
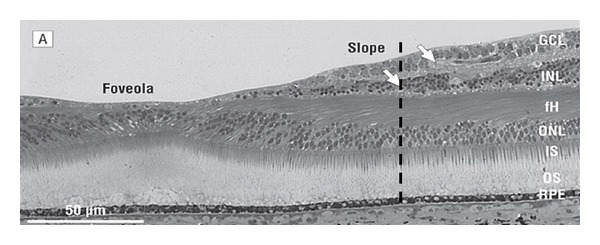
This shows histology of the human retina (after Provis and Hendrickson). Interrupted lines represent perifoveolar radial distances of 0.9 and 1.25 mm where ganglion cells begin to dominate inner retinal thickness. Maximal IA occurs at distances less than 0.9 mm [22].

The tables show that the highest number of patients can be best distinguished at 0.5 and 0.75 mm radial distance.  Table 5 represents the grouped data points for 0.5 and 0.75 mm at 1 SD in a predictive-value plot. The inner retina at this particular distance contains some ganglion cells and mostly inner plexiform elements. It represents the zone of the foveal slope, where ganglion cells begin to emerge (see Figure 3). 

A second flatter “peak” may be present at 1.75 to 2 mm radial distance where ganglion cells and the NFL begin to make a dominant contribution (see Figure 3, after Provis and Hendrickson) [22] to retinal thickness.

**Figure 4:**
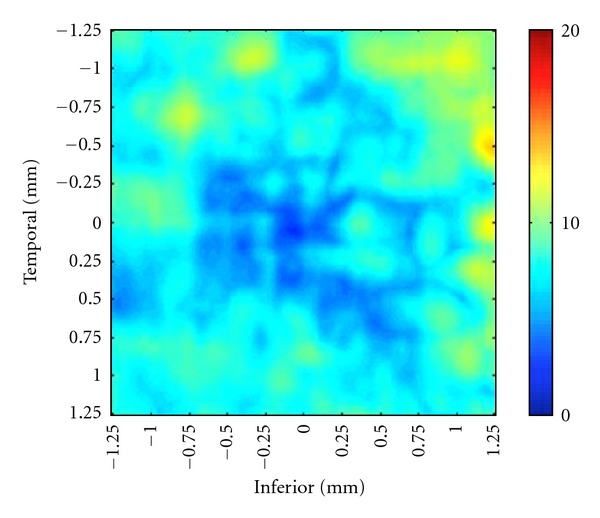
The mean interocular difference map of all control subjects is depicted. Thickness measurements of the inner retina over the central grid of 2.5 × 2.5 mm were obtained, digitally reconstructed, and color-coded. Temporal side is on the left and nasal retina on the right side. Left eyes were reflected horizontally so that the temporal retina is directionally left and the nasal retina is directionally right.

Apparently both foveal slope architecture and ganglion cell thickness contribute to deviant IA in PD. By taking 1.5 SD as the criterion, we find that as a population PD patients show significant interocular difference only at radial distances of 0.5, 0.75, and 1 mm from the foveola. Interestingly, this corresponds to the slope of the foveal pit. For a 2 SD criterion, however, there is little IOD.  Figure 4 shows the mean interocular difference for all control subjects.

## 4. Discussion

PD patients have greater IOD in retinal thickness than controls. There appears to be an important difference corresponding to the foveal pit in PD. In the foveal pit, inner but not full-foveal thickness seems to discriminate between PD and controls. Furthermore, interocular symmetry of retinal thickness varies with perifoveolar distance in the fovea in PD. IA appears most evident (see Figure 2) at some distance from the foveola at the slope of the foveal pit where ganglion cells are still scarce [19]. Neither our data nor previous evidence suggests that race, gender, age, and axial length have significant effect on IA of the foveal pit (see Supplementary Material 4). Measuring average macular volume, easy to execute on all OCT models, includes all the inner and outer retinal layers. The distance at 0.75 mm defines a diameter of 1.5 mm. For this diameter, the ETDRS volume did not discriminate between PD and controls. It is likely that the difference for the volumetric measure was not significant as the volume included other regions, closer to the foveola, and outer retinal layers. Our results suggest that therefore macular volumes need to be treated with caution sincepathology (as occurs in PD) may not equally affect diverse layers in the foveal pit.

In studies of eye disorders, it is important to consider, for statistical comparison and diagnostic yield, the number of eyes versus the number of subjects [20, 21]. If the correlation between studied variables in the two eyes is high (when within subject variability is low), then it would be permissible, in a large population to use one eye data only. However, a perfect correlation depends on the measure selected. For instance, the interocular correlation of diurnal variation and intraocular pressure (IOP) [25] are highly similar in the two eyes of subjects; however, the coefficients of determination for single pairs range from 0.311 to 0.741. We recommend evaluating each eye of each patient in PD. A clinicaladvantage of quantifying interocular symmetry is that it is less dependent on absolute thickness measurements and thus less dependent on equipment differences [26].

Motor asymmetry is part of the criteria for clinical and imaging [27, 28] diagnosis of PD. If a patient exhibits symmetrical motor findings, the diagnosis of PD must necessarily be questioned. Although motor asymmetry is clinically well accepted, the reasons for this asymmetry are not well understood [29]. It was suggested that asymmetry is a random process, while dominant handedness [30] is associated with motor asymmetry. Eye dominance and interocular asymmetry in PD have not been evaluated. As the neurosensory retina is impacted in PD, it is plausible that the earliest changes are also asymmetrical and that an area of specificity for the disease would vary amongst a person's eyes. It would thusly be useful as a screening tool and marker of disease presence and progression. 

In the search for a quantitativeophthalmological tool to be used as a biomarker for PD (i.e., SD-OCT), a larger normative database is needed. Neurodegenerative diseases increase with age and a number of them have been shown to affect the retina. Both our patients and controls were selected based on identical and rigorous ophthalmological and neurological in- and exclusion criteria (see Supplementary Table 1). We are not aware of any relevant published OCT study in which controls would have been thoroughly screened for neurological conditions. In the future, multivariate analysis may be useful for relaxing the strict criteria to include patients with common conditions such as diabetes mellitus, for instance, who were excluded in the present study.

## 5. Conclusions

We demonstrate that IA measured at certain radial peri-foveal distance may provide help in discriminating between normal and PD subjects. This discrimination affected only about 1 out of five patients, but the specificity is high. Discrimination appears to be optimal at perifoveal distances of 0.5 and 0.75 mm. This distance corresponds to a region on the slope of the foveal pit where the ganglion cells just begin to emerge. A second flatter “peak” could be present at 2 mm radial distance where ganglion cells and the NFL begin to make a dominant contribution to retinal thickness. Apparently both foveal slope architecture and ganglion cell thickness contribute to deviant IA in PD. IA of the foveal shape has a high specificity and may be a potentially useful marker for PD and may be useful in large multicenter clinical trials.

## Supplementary Material

Supplement to methods in the manuscript

## Figures and Tables

**Table 1:** Interocular thickness difference (microns) and SDs (1, 1.5, and 2) by radial distance location (mm from foveola) in PD patients and controls.

	0.25 (mm)	0.5 (mm)	0.75 (mm)	1.0 (mm)	1.25 (mm)	1.5 (mm)	1.75 (mm)	2.0 (mm)
Control (mean)	5.50	6.20	5.53	6.97	6.61	7.23	6.32	5.71
PD (mean)	5.11	8.54	10.17	12.26	11.92	10.49	9.76	9.77
Control 1 SD	4.90	4.83	5.54	7.39	8.82	8.69	6.66	6.65
Control 1.5 SD	7.35	7.24	8.32	11.08	13.24	13.04	9.99	9.97
Control 2 SD	9.80	9.66	11.09	14.78	17.66	17.39	13.32	13.30

**Table 2:** Individual subjects outside their group's retinal thickness mean IOD at 1 control SD at each radial distance (mm). It is evident that the most false positives (controls) were at 1 and 1.25 mm. The highest percentage of correctly identified patients was at 0.5 and 0.75 and to a lesser extent at 1 mm and 1.75 perifoveolar distance (see also Table 5).

	0.25 (mm)	0.5 (mm)	0.75 (mm)	1.0 (mm)	1.25 (mm)	1.5 (mm)	1.75 (mm)	2.0 (mm)
Number of Ctrl	2	1	0	1	2	3	1	1
Number of PD	0	4	5	3	2	3	4	1
Percentage of 23 PD patients	0	17.4	21.7	13	8.7	13	17	4.3
Percentage of 18 controls	11.1	5.5	0	5.5	11.1	16.7	5.5	5.5

**Table 3:** Individual subjects outside their group's retinal thickness mean IOD at 1.5 control SD at each radial distance (mm).

	0.25 (mm)	0.5 (mm)	0.75 (mm)	1.0 (mm)	1.25 (mm)	1.5 (mm)	1.75 (mm)	2.0 (mm)
Number of Ctrl	0	0	0	0	1	2	1	1
Number of PD	0	2	4	1	2	2	1	0
Percentage of 23 PD patients	0	8.7	17.4	5.6	8.7	8.7	5.6	0
Percentage of 18 controls	0	0	0	0	5.5	11.1	5.5	5.5

At the stricter (1.5 SD) criterion, 0.75 mm remains as the optimal distance for discriminating PD and controls (compare this table to Tables 1 and 2).

**Table 4:** Individual subjects outside their group's retinal thickness mean IOD at 2 control SD at each radial distance (mm).

	0.25 (mm)	0.5 (mm)	0.75 (mm)	1.0 (mm)	1.25 (mm)	1.5 (mm)	1.75 (mm)	2.0 (mm)
Number of Ctrl	0	0	0	0	1	0	1	1
Number of PD	0	1	1	1	1	1	1	0
Percentage of 23 PD patients	0	4.3	4.3	4.3	4.3	4.3	4.3	0
Percentage of 18 controls	0	0	0	0	5.5	0	5.5	5.5

**Table 5:** Predictive value table for grouped data points at 0.5 and 0.75 mm radial distance (1 SD).

		Significant IOD	
		−	+
PD	−	98.2 %	2.8 %
	+	80.4 %	19.6 %
