# Supplementary material for: Interocular Asymmetry of Foveal Thickness in Parkinson Disease
Source: J Ophthalmol. 2012 Aug 1;2012:728457. doi: 10.1155/2012/728457 (PMC3415246; doi:10.1155/2012/728457)
Supplement: Supplementary file 1 — Supplement to methods in the manuscript [file 728457.f1.pdf]

Supplementary 1:

The first evidence of visual impairment in PD and its relationship to dopaminergic dysfunction was obtained by Bodis-Wollner and Yahr.[1] Utilizing Visual Evoked Potential (VEP) measurements they noted asymmetrical delay of the VEP obtained from the two eyes in PD. Subsequently Bodis-Wollner, Yahr and Thornton[2] reported the conjoint interocular statistical correlation of VEP abnormality with PD staging (H-Y). A number of studies using the ERG identified impaired retinal ganglion cell processing as a reason for visual impairment in PD.[3,4,5,6,7,8] Most recently it was shown that nerve-fiber layer (NFL) thinning correlates with the multifocal ERG in PD and confirms an association with retinal ganglion cell processing, similar to defects seen in glaucoma.[9] However, an interocular, within-subjects comparison of retinal thickness was not considered quantitatively in any previous OCT study in PD.

Supplementary 2:

The retinal area of our measurements and OCT:

Through SD-OCT, morphology felt to be closely representative of gross retinal histology can be measured, imaged and reproduced.[10] The fovea, is an area approximately 1.5 mm in radius.[11,12] The very center of the fovea is called the foveola. Ganglion cells and their axons (nerve fibers) and other inner retinal neuronal elements are pushed aside and are hence absent from the foveola. There are three major forward synapses before the ganglion cells in the innermost layer receive the signals of receptors.

Supplementary 3:

Subject selection criteria for PD patients and control:

Neurological Inclusion criteria: (1). Able and willing to give informed consent and comply with study protocol. (2). Idiopathic Parkinson Disease, based on the UK Brain Criteria[13], UPDRS and Hoehn and Yahr scale.[14]

Neurological Exclusion criteria: Young onset (<40 years of age) PD, no response to levodopa/dopamine agonist, after a sufficient trial of these medications (at least 3 weeks of 600 mg of L-dopa or equivalent doses of dopamine agonists), Mini-mental state examination (MMSE) score below 26, brain imaging abnormalities suggestive of other cause for Parkinsonism, unusual or atypical risk factors (i.e. significant illicit drug exposure, acute or chronic toxin exposure, severe or repetitive head trauma), strong family history of PD; prior stroke.

Neurological Exclusion criteria for being a control subject: Any signs of neuro-degenerative disease (i.e. abnormal motor findings or MMSE score below 26).

Specific Opththalmic Exclusion criteria (PD and controls): (1). A personal history of Diabetes mellitus (2). Significant cataract (3). Intraocular pressure measurement > 18 mm Hg or the presence of anatomically "narrow anterior chamber angles", glaucoma or glaucoma suspicion of any type (4). Any prior intraocular surgical procedure or laser treatment (6). Personal or a strong family history of a retinal degeneration/dystrophy (7). High degree of myopia (> 8 diopters) or hyperopia (>4 diopters) (8). Optic nerve abnormality (9). or any past or evident retinal vascular disease.

Supplementary 4:

Effects of race, gender, age and axial length on the IA of the fovea.

Macular volume measures

A number of OCT studies considered changes in full-retinal thickness (FRT) as a result of age, gender, race and axial length but not all came to the same conclusions. El-Ashry et al.[15] quantified minimum foveal thickness (MFT), central 1 mm average foveal thickness (AFT) and total macular volume in 100 British healthy individuals. AFT was symmetrical between the eyes of each individual and there was no effect of age, gender or race on interocular differences. In 110 eyes of 57 subjects including Europeans and Afro-Caribbeans Tick et al.[16] reported a high degree of symmetry in all examined morphometric parameters (CFT, pit depth, pit diameter and MRT). We found no interocular asymmetry difference in our, partially overlapping measures of foveal architecture. Not all but a number of studies of slope, depth and rim of the fovea came to the conclusion, that blacks have thinner retina than Caucasians.[17,18,19]

Race was not significant as a main effect in our study, however there were absolute differences and race nearly reached statistical significance. We believe therefore that it is worthy of further studies to examine whether race affects primarily ORL's, including Henle's fiber layer, Bruch's membrane and the RPE. Axial length correlates with pit diameter[15] however the causality is unclear except perhaps in patients with high myopia; thusly we excluded that condition from both the control and PD groups. There is no evidence that PD primarily changes the axial length of the eye, but as a result of foveal

pit changes, axial length may potentially change in PD. This possibility requires further study. Age is not a primary variable in our study: there was insignificant age difference between the subjects. IO was not studied at discrete peri-foveolar distances and no previous information is available for the effects of age, race and gender. Our results show in PD there is a perifoveolar distance dependent effect on the inner neural layers of the foveal pit. Given the different cellular composition of various layers of the foveal retina, we do believe that quantifying the effect of these variables, segmenting across the inner and outer retinal layers and perifoveolar distances would be meaningful.

Supplementary references:

1. Bodis-Wollner I and Yahr MD. Measurement of visual evoked potentials in Parkinson's disease. *Brain.* 1978;101:66l-67l.

2. Bodis-Wollner I, Yahr MD, Thornton J. Visual evoked potentials and the severity of Parkinson disease. In: Research Progress in Parkinson's disease, *C Rose and R Capildeo (eds) Tunbridge Wells: Pitman Medical*:126-137.

3. Gottlob I, Schneider E, Heider W, Skrandies W Alteration of visual evoked potentials and electroretinograms in Parkinson's disease. *Electroencephalogr Clin Neurophysiol.* 1987 Apr;66(4):349-57.

4. Ikeda H, Head GM, Ellis CJ. Electrophysiological signs of retinal dopamine deficiency in recently diagnosed Parkinson's disease and a follow up study. *Vision Research.* 1994;34:2629-38.

5. Calzetti S, Franchi A, Taratufolo G, Groppi E. Simultaneous VEP and PERG investigations in early Parkinson's disease. J Neurol Neurosurg Psychiatry. 1990; 53(2):114-117.

6. Tagliati M, Bodis-Wollner I, Yahr M. The pattern electroretinogram in Parkinson's disease reveals lack of retinal spatial tuning. Electroenceph and Clin Neurophysiol. 1995;100:1-11.

7. Peppe A, Stanzione P, Pierantozzi M, et al. Does pattern electroretinogram spatial tuning alteration in Parkinson's disease depend on motor disturbances or retinal dopaminergic loss? *Electroencephalogr Clin Neurophysiol.* 1998;106(4):374-382.

8. Sartucci F, Orlandi G, Lucetti C, et al.Changes in pattern electroretinograms to equiluminant red-green and blue-yellow gratings in patients with early Parkinson's disease. *J Clin Neurophysiol*. 2003;20 (5):375-381.

9. Moschos MM, Tagaris G, Markopoulos I, et al. Morphologic changes and functional retinal impairment in patients with Parkinson disease without visual loss. *Eur J Ophthalmol.* 2011; 21(1):24-29.

10. Wojtkowski M, Bajraszewski T, Gorczyñska I, et al. Ophthalmic imaging by spectral optical coherence tomography. *Am J Ophthalmol. 2004;*138:412–419.

11. Huang D, Swanson EA, Lin CP, et al. Optical coherence tomography. *Science.* 1991;254:1178-1181.

12. Provis JM, Hendrickson AE. The foveal avascular region of developing human retina. *Arch Ophthalmol.* 2008;126:507-511.

13. Hughes AJ, Daniel SE, Kilford L, Lees AJ. Accuracy of clinical diagnosis of idiopathic Parkinson's disease: a clinic-pathological study of 100 cases. *J Neurol Neurosurg Psychiatry. 1992;*55:181-184.

14. Hoehn MM, Yahr MD. Parkinsonism: onset, progression and mortality. *Neurology. 1967;*17:427–442.

15. El-Ashry M, Hegde V, James P, Pagliarini S. Analysis of macular thickness in British population using optical coherence tomography (OCT): an emphasis on interocular symmetry. *Curr Eye Res. 2008;*33**:**693-699.

16. Tick, S, Rossant, F, Ghorbel, I, at al. Foveal Shape and Structure in a Normal Population. *Invest Ophthalmol Vis Sci.* 2011;52(8):5105-5110.

17. Wagner-Schuman M, Dubis AM, Nordgren RN, et al. Race- and sex-related differences in retinal thickness and foveal pit morphology. *Invest Ophthalmol Vis Sci*. 2011;52(1):625-34.

18. Song WK, Lee SC, Lee ES, et al. Macular thickness variations with sex, age, and axial length in healthy subjects: a spectral domain-optical coherence tomography study. *Invest Ophthalmol Vis Sci*. 2010;51:3913–3918.

19. Kim, NR, Kim, JH, et al. Determinants of perimacular inner retinal layer thickness in normal eyes measured by Fourier-domain optical coherence tomography. *Invest Ophthalmol Vis Sci.* 2011; [Epub ahead of print].

Supplementary Table 1: Questionnaire for Ocular Exclusion

		Yes	No	Unsure	***ANSWERS***
**1**	Have you ever had a comprehensive eye exam **including dilation**?	Y	N	?	
**2**	Have you ever been diagnosed with Glaucoma?	Y	N	?	
**3**	Do you have a family history of Glaucoma?	*y*	*n*	*?*	
**4**	Have you ever been diagnosed with Diabetes, even borderline?	Y	N	?	
**5**	Do you have any personal history of Age Related Macular Degeneration?	Y	N	?	
**6**	Are you highly myopic (nearsighted), with a prescription power of ≥ **-6.50 D**?	Y	N	?	
**7**	Have you ever been treated for an eye disease involving the middle of the eye? (e.g., iritis, uveitis, vitritis)	Y	N	?	
**8**	Have you ever been diagnosed with Pseudoexfoliation Syndrome?	Y	N	?	
**9**	Have you ever been diagnosed with Pigment Dispersion Syndrome?	Y	N	?	
**10**	Have you ever been told you have "Narrow Angles"?	Y	N	?	
**11**	Have you ever been informed of any lesion(s) on your retina? If yes, then:	*y*	*n*	*?*	
**(a)**	was the lesion a Choroidal Nevus?	*y*	*n*	*?*	
**(b)**	was the lesion inflammatory?	Y	N	?	
**12**	Do you have any history, *including family history,* of retinal degeneration or dystrophy?	Y	N	?	

**13**	Do you have any history, *including family history,* of optic neuropathy/ atrophy?	Y	N	?	
**14**	Do you have any personal history of optic nerve head drusen?	Y	N	?	
**15**	Do you have any personal history of optic neuritis? (Including retro-bulbar optic neuritis and papillitis?)	Y	N	?	
**16**	Do you have any personal history of hypertensive retinopathy (retinal bleeding due to extremely high blood pressure)?	Y	N	?	
**17**	Have you ever had any eye surgery, laser surgery, or intraocular injections?	*y*	*n*	*?*	
**18**	Was the eye surgery cosmetic, or limited to the eyelids?	*y*	*n*	*?*	
**19**	Was the (non-cosmetic) eye surgery **less than 90 days ago**?	Y	N	?	
**20**	Did you ever have a peripheral iridotomy? (Drainage hole lasered through the iris)	Y	N	?	
**21**	Did you ever have cataract surgery?	*y*	*n*	*?*	
	Right eye? If yes, **date:** ______	*y*	*n*	*?*	
	Left eye? If yes, **date:** ________	*y*	*n*	*?*	

Supplementary Table 1 (continued):
